# Oral delivery of *Eimeria acervulina* transfected sequentially with two copies of the VP2 gene induces immunity against infectious bursal disease virus in chickens

**DOI:** 10.3389/fvets.2024.1367912

**Published:** 2024-04-10

**Authors:** Qingbin Guo, Ying Yu, Jingxia Suo, Xinming Tang, Sixin Zhang, Colin Crouch, Beth Bruton, Ian Tarpey, Xianyong Liu, Guanghui Zhao, Xun Suo

**Affiliations:** ^1^College of Veterinary Medicine, Northwest A&F University, Xianyang, China; ^2^National Animal Protozoa Laboratory and College of Veterinary Medicine, China Agricultural University, Beijing, China; ^3^Key Laboratory of Animal Biosafety Risk Prevention and Control (North) of MARA, Institute of Animal Science, Chinese Academy of Agricultural Sciences, Beijing, China; ^4^MSD Animal Health, Milton Keynes, United Kingdom

**Keywords:** chicken, coccidia, delivery vector, genetic manipulation, viral antigen

## Abstract

Chicken coccidiosis caused by *Eimeria* spp. can occur on almost all poultry farms, causing huge economic losses to the industry. Genetically manipulated *Eimeria* parasites as a vaccine vector to deliver viral antigens have been reported. In our preliminary study, transgenic *E. acervulina* expressing a VP2 gene (Ea-VP2) of the infectious bursal disease virus (IBDV) demonstrated partial protection against IBDV infection. To enhance immune responses, we aimed to increase the VP2 gene copy number in transgenic *E. acervulina*. In this study, we used a novel plasmid vector carrying a VP2 gene fused with three flag tags and a red fluorescent reporter gene (mCherry). The vector was introduced into Ea-VP2 sporozoites through nucleofection, leading to the generation of Ea-2VP2. Subsequent analysis revealed a notable escalation in the fluorescent rate, increasing from 0.11 to 95.1% following four consecutive passages facilitated by fluorescent-activated cell sorting. Verification via PCR, Western blot, and immunofluorescence confirmed the successful construction of the Ea-2VP2 population. Despite lower fecundity compared to wild-type *E. acervulina*, Ea-2VP2 maintained immunogenicity. Our research effectively created a transgenic *E. acervulina* strain transfected sequentially with two copies of the VP2 gene from IBDV. This modification resulted in an increased humoral immune response after primary immunization in chickens. Additionally, it demonstrated a degree of protection within the bursa against IBDV infection. Future studies will focus on further enhancing immune response levels.

## 1 Introduction

Chicken coccidiosis is an enteric disease and causes more than £10.4 billion (according to 2016 prices) of economic cost to the chicken industry globally each year (1). Current live anti-coccidial vaccines are reliable to induce an efficient and long-lasting immune response for the prevention and control of chicken coccidiosis (2, 3). Live anti-coccidial vaccines can be delivered through drinking water and feed (4). However, live anti-coccidial vaccines are species- or even strain-specific, so effective anti-coccidial vaccines must include multiple *Eimeria* species or strains and need to be produced independently in chickens, requiring further refinements (4, 5).

The pathogen is a eukaryotic single-celled protozoan belonging to the genus *Eimeria* (6). *Eimeria* parasites have 14 chromosomes with 42–72 Mbp DNA in length, encoding 6,000 to 9,000 proteins across all developmental stages (7). So far, 10 species of chicken coccidian have been clearly identified, including *E. acervulina*, *E. tenella*, *E. maxima*, *E. mitis*, *E. necatrix*, *E. brunetti*, *E. praecox*, *E. lata*, *E. nagambie*, and *E. zaria* (8). Among these, *E. acervulina* is highly prevalent but has moderate pathogenicity (9). One *Eimeria* sporozoite can yield 1,000 merozoites per cycle, repeating two to four times in the chicken host, leading to a rapid surge in oocyst numbers (10). Chicken coccidians are highly immunogenic, and their primary infection triggers protective immunity against subsequent homologous parasite infections (11). Moreover, chicken coccidians enter host cells aided by their microneme protein, easily prompting immune responses to carried viral proteins upon oral administration, showcasing more potential as an oral vaccine delivery vector (12, 13).

With the ongoing progress in bioinformatics and genetic manipulation, *Eimeria* parasites have been considered a vector to express exogenous antigens (14). Research findings indicate that a transgenic population of *E. tenella* expressing the *Campylobacter jejuni* antigen CjaA (*E. tenella*-CjaA), following either single or multiple oral vaccinations with *E. tenella*-CjaA, provides immune protection rates of 91 and 86% against subsequent *C. jejuni* challenges, and these rates are significantly higher compared to unvaccinated and wild-type *E. tenella*-vaccinated groups in chickens (15). Moreover, orally inoculating with transgenic *E. tenella* expressing two antigen genes of *Eimeria maxima* (EmIMP1 or EmAMA1) can significantly reduce oocyst production after the *E. maxima* challenge (16, 17). In a separate investigation, *E. tenella* was used as a vector expressing viral antigens from either infectious bursal disease virus (IBDV) or infectious laryngotracheitis virus (ILTV), but the detection of antibodies via Western blot was only successful when using low-dilution serum (13).

IBDV instigates a critical immunosuppressive disease, proving lethal to chickens aged 3–6 weeks by destroying immature B lymphocytes and diminishing immune capabilities (18–20). The IBDV genome comprises two double-stranded RNA (dsRNA) segments, A and B, housing five genes: VP1, VP2, VP3, VP4, and VP5 (21, 22). VP2, constituting the capsid, serves as the primary target for antiviral-neutralizing antibodies (23). Various expression systems have been used to produce the VP2 protein, such as yeast and lactobacillus (24, 25). In our preliminary investigation, transgenic *E. acervulina* expressing a single VP2 was able to induce an immune response (26). However, enhancing immune response levels remains imperative for effective control of IBDV.

In this study, we hypothesized that increasing the number of heterologous antigens in transgenic *E. acervulina* could enhance the protective immune response. To address the hypothesis, we constructed a novel plasmid, including a VP2 gene and a red fluorescent reporter gene (mCherry), and then transfected with the plasmid into the sporozoites which have been stably transfected a plasmid carrying a VP2 gene with an enhanced yellow fluorescent reporter gene (EYFP). Subsequently, we performed a series of experiments to identify the expression of the exogenous VP2 gene in the transgenic *E. acervulina* and studied the endogenous developmental stage, fecundity, and immunogenicity. In addition, we detected the humoral immune response and evaluated the protection against pathology in the bursa caused by IBDV.

## 2 Materials and methods

### 2.1 Animals, parasites, and cell culture

Specific pathogen-free (SPF) chickens, 1 week old, were purchased from Boehringer Ingelheim Biotech Limited (Beijing, China). Arbor Acres (AA) broilers, 1–6-weeks-old, were purchased from Arbor Acres Poultry Breeding (Beijing, China). All chickens were kept in poultry-specific isolators and fed with coccidia-free water and feed.

The wild-type *E. acervulina* (Ea-WT) Beijing strain came from our laboratory at China Agricultural University. Transgenic *E. acervulina* expressing a single VP2 gene of IBDV (Ea-VP2) was also maintained, and the VP2 and microneme 2 genes of *E. acervulina* (EaMic2) genes were genetically linked via a flexible glycine–serine linker (L14) (26). Oocysts were collected from feces, passaged, sporulated, and purified according to previous procedures and methods (27).

Human foreskin fibroblast (HFF) cells were purchased from the American Type Culture Collection (ATCC) and cultured in Dulbecco’s Modified Eagle Medium (DMEM) medium with fetal bovine serum (10% v/v) and 1000 U streptomycin–penicillin in a cell culture incubator with 5% CO_2_ at 37°C.

### 2.2 Plasmid construction and stable transfection of *Eimeria acervulina*

**Figure 1:**
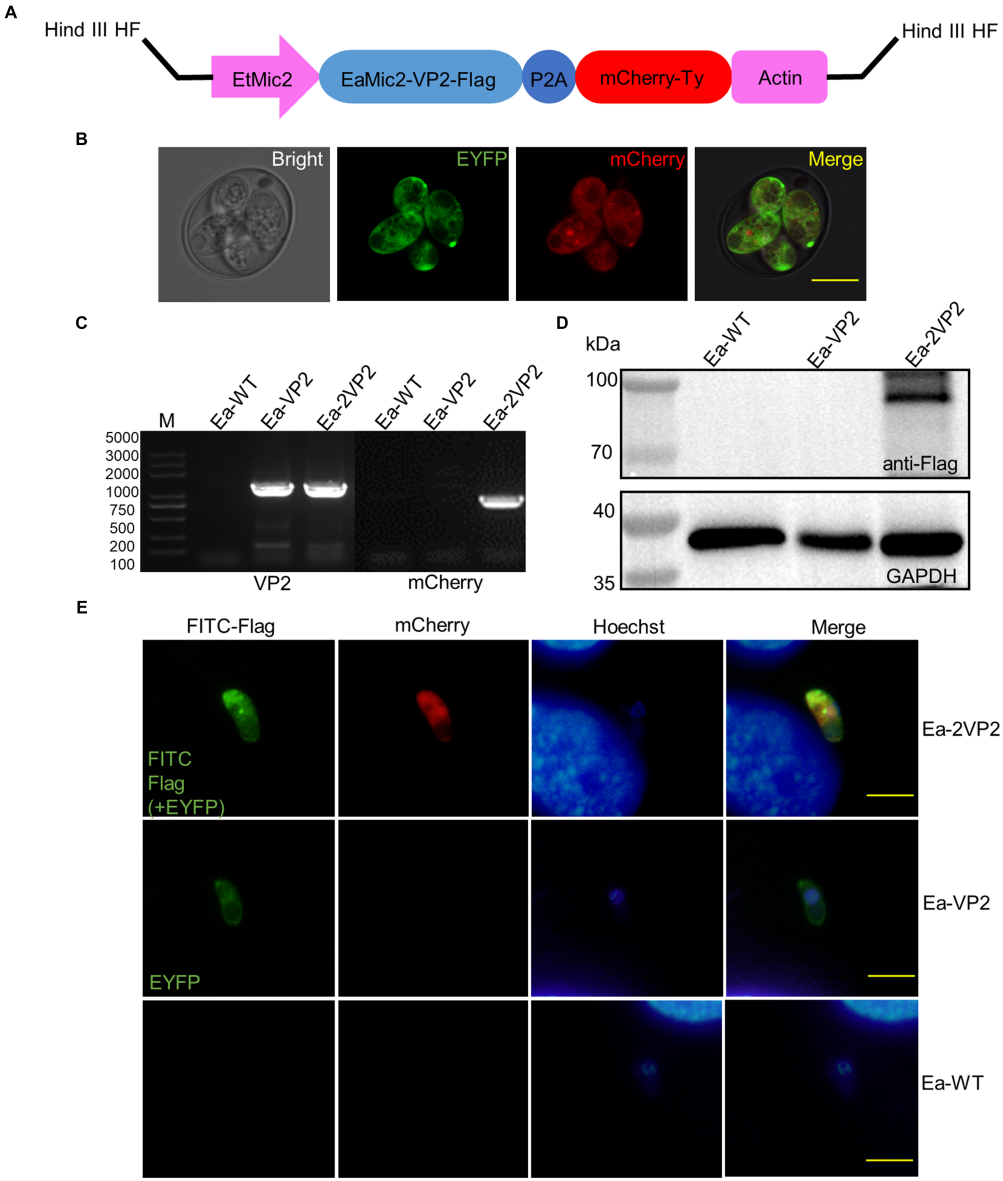
Construction of transgenic *E. acervulina* transfected sequentially with two copies of the VP2 gene of IBDV (Ea-2VP2). **(A)** Scheme or diagram of expression vector linearized by *Hind* III HF restriction enzyme. **(B)** The stable transfected Ea-2VP2 expresses EYFP and mCherry in a sporulated oocyst. **(C)** Genomic DNA from Ea-2VP2 was amplified with the primers VP2 and mCherry, giving a 1,359 bp and 708 bp product, and the genomic DNA from Ea-WT and Ea-VP2 were used as controls. **(D)** Western blot analysis of the expression of EaMic2-VP2-flag fused protein in Ea-2VP2. The mouse anti-flag monoclonal antibody 1804 (1:2000) was used as the primary antibody, and the soluble proteins from Ea-WT and Ea-VP2 were used as negative controls. **(E)** Cellular localization patterns of VP2 in the sporozoites of Ea-2VP2 by IFA. The mouse anti-flag monoclonal antibody 1804 (1:200) was used as the primary antibody, and the sporozoites from Ea-WT and Ea-VP2 were utilized as a control. Nuclei were stained with hoechst. Bar = 10 μm.

A novel plasmid, pMic-VP2-flag-mCherry-Actin, was constructed based on previous plasmids constructed in our laboratory. The VP2 fragment sequence of IBDV was kindly provided by MSD Animal Health, codon-optimized, and synthesized by Beijing Tsingke Biotechnology Co. Ltd. VP2 and EaMic2 genes were genetically linked with three flag tags via L14. The red fluorescent reporter gene (mCherry) with ty tags was linked by a porcine teschiovirus-1 2A peptide (P2A, 66 bp) (28). These elements were regulated by a microneme 2 promoter of *E. tenella* (EtMic2), such as Ea-VP2. The circular DNA plasmid was constructed using a seamless cloning kit (TransGen Biotech, China) and then linearized using the *Hind* III HF restriction enzyme (Figure 1A). As shown in Supplementary Table 1, primers are used for the cloning of the different regions of the plasmid.

For the stable transfection of *E. acervulina*, 10 μL of linearized DNA plasmid and 5 μL of *Hind* III HF were transfected into 1 × 10^7^ sporozoites of Ea-VP2 using restriction enzyme-mediated nuclear transfection (Program U-033, AMAXA, Switzerland) (29). Then, the sporozoites were injected into four 3-week-old chickens via the wing vein, and oocysts were collected in the feces from days 5–8 after inoculation. The transfected oocysts successfully expressing EYFP and mCherry were selected by fluorescent-activated cell sorting (FACS) (DakoCytomation, Fort Collins, CO), and then orally inoculated into coccidia-free chickens for passage for the selection of transfected parasites. The purified oocysts were stored in 2.5% KCr_2_O_7_ at 4°C.

### 2.3 Genomic DNA identification of transgenic *Eimeria acervulina*

Genomic DNA was extracted from sporulated transgenic oocysts according to the previous description (29). PCR primer pairs (forward and reverse primer sequences) targeting VP2 and mCherry genes were VP2-F/VP2-R and mCherry-F/mCherry-R (Supplementary Table 2). The VP2 and mCherry genes were amplified using the above total genomic DNA as templates. The PCR products were confirmed by DNA sequencing. Genomic DNA samples from Ea-VP2 and Ea-WT were used as controls.

### 2.4 Western blot analysis

Protein was extracted from sporulated transgenic oocysts as previously described (30). Soluble protein was separated by sodium dodecyl sulfate-polyacrylamide gel electrophoresis (SDS-PAGE) and electrotransferred onto polyvinylidene difluoride (PVDF) membranes. The fusion protein (EaMic2-VP2-flag) was detected using commercial mouse anti-flag monoclonal antibody 1804 (1:2000 dilution) probed on PVDF membranes at 37°C for 1 h. Detection was carried out with an enzyme-labeled goat anti-mouse secondary antibody (1:2000 dilution) probed at 37°C for 1 h. The glyceraldehyde-3-phosphate dehydrogenase (GAPDH) of *E. acervulina* was used as an internal reference. An enhanced chemiluminescence solution was applied to the PVDF membranes and then placed into the Tanon instrument for development. The theoretical molecular weight of the target protein contains VP2 (49.83 kDa), EaMIC2 (32.23 kDa), and three flag tags (2.42 kDa). Proteins from Ea-VP2 and Ea-WT were used as controls.

### 2.5 Indirect immunofluorescence assay

To validate the localization of VP2 protein in the transgenic Ea-2VP2 sporozoites, HFF cells were infected with 1 × 10^6^ sporozoites of Ea-2VP2 in a cell culture incubator for 6 h. Briefly, the sporozoites were fixed in 4% paraformaldehyde for 1 h at 37°C and then permeabilized with 0.25% Triton X-100 for 20 min, followed by blocking with 3% bovine serum albumin (BSA) for 15 min. Mouse anti-flag monoclonal (1:200) was used as the primary antibody to detect the fusion protein (EaMic2-VP2-flag), followed by FITC-conjugated goat anti-mouse IgG (1:200) as the secondary antibody at 37°C for 1 h. After staining the nuclei with Hoechst 33258, the slides were sealed with antifade mounting media and observed using a fluorescent microscope (IX71, Olympus). Sporozoites from Ea-VP2 and Ea-WT were used as controls.

### 2.6 H&E and tissue immunofluorescence

The duodenum of 1-week-old SPF chickens was sampled during 48–120 h with an interval of 12 h after infection with 5 × 10^5^ Ea-2VP2. The sampled duodenums were fixed in 4% neutral-buffered formalin for 48 h, embedded in paraffin, and stained with hematoxylin and eosin (H&E). H&E staining and tissue sections were prepared for tissue immunofluorescence as previously described (31). Briefly, tissue sections must be first deparaffinized with xylene and washed in serial dilutions of ethanol, then antigen repair, immediately after which the slides were added with 3% BSA into the circle and covered the tissue evenly to block non-specific binding at room temperature for 30 min. Mouse anti-flag monoclonal (1:200) was used as the primary antibody to detect the fusion protein (EaMic2-VP2-flag), followed by FITC-conjugated goat anti-mouse IgG (1:200) as the secondary antibody for 1 h. The nuclei were stained with DAPI for 7 min, and then slides were observed with a fluorescent microscope.

### 2.7 Measurement of the fecundity and immunogenicity of Ea-2VP2

Three groups of three 1-week-old SPF chickens were individually caged in poultry-specific isolators and orally inoculated with 500 freshly sporulated oocysts of Ea-2VP2, Ea-VP2, and Ea-WT, respectively. Oocysts were collected from feces and counted using a McMaster chamber with an interval of 24 h, from days 3 to 14 post-inoculation (DPI). Chickens in all three groups were orally challenged with 5,000 freshly sporulated oocysts of Ea-WT on day 14. Afterward, oocysts were collected in the feces from days 5 to 7 post-challenge (DPC); the Ea-VP2 group and the Ea-WT group served as controls.

### 2.8 Immunizations and challenge experiment

**Figure 2:**
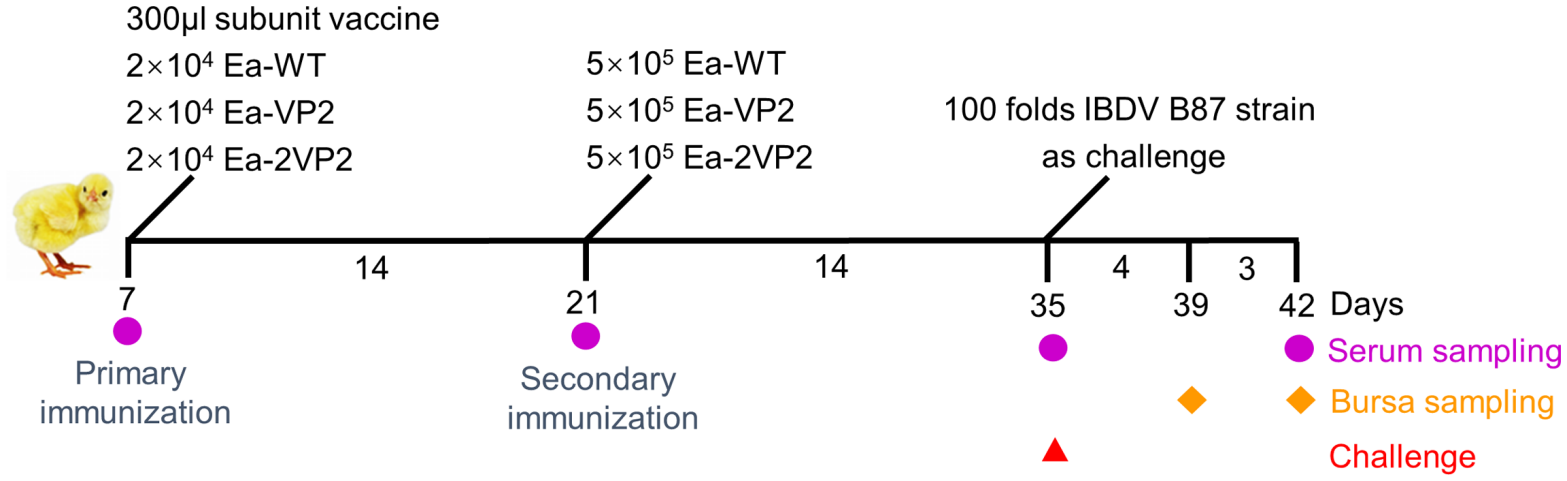
Procedures for immunizations, challenges, and tissue sampling during animal experiments. At 7 and 21 days of age, SPF chickens received oral primary immunization and secondary immunization, respectively. Immunizations were conducted two times at intervals of 2 weeks. On day 14, after the secondary immunization, chickens were challenged with the 100-fold IBDV B87 strain as prescribed, except for the UUC group. Serum samples were collected at 7, 21, 35, and 42 days of age. Five chickens were randomly taken from each group at 39 and 42 days of age to examine the bursa.

Seven-day-old SPF chickens were randomly divided into 6 groups (20 chickens per group) (Supplementary Table 3). The immunization scheme and sample collection were shown on a time axis (Figure 2). Unimmunized and unchallenged control (UUC) group, unimmunized and challenged control (UCC) group, commercial vaccine (a quadruple vaccine, containing protein rVP2 of IBDV) (vaccine) group, Ea-WT group, Ea-VP2 group, and Ea-2VP2 group were designed. The primary immunization was administered at 7 days of age; the vaccine group was administered intramuscularly with the prescribed dose (300 μL/per chicken); the Ea-WT, Ea-VP2, and Ea-2VP2 groups were each inoculated with 2 × 10^4^ freshly sporulated oocysts. The secondary immunization was carried out at 21 days old; the Ea-WT, Ea-VP2, and Ea-2VP2 groups were each inoculated with 5 × 10^5^ freshly sporulated oocysts, respectively. At 35 days of age, except for the UUC group, the rest of the groups were all challenged with the IBDV B87 strain 100-fold as prescribed.

### 2.9 Enzyme-linked immunosorbent assay (ELISA)

For serological tests, the blood was collected at 7, 21, 35, and 42 days of age (Figure 2). Bloods were incubated at 37°C for 1 h, then transferred to 4°C for 2 h, and serum was collected after centrifugation at 5000 rpm for 10 min and stored at −20°C until further use. The presence of serum antibodies against the VP2 antigen of IBDV was detected using a commercial ProFLOK^™^ IBD Plus antibody test kit (Zoetis, China). When the antibody titer was >999, it was considered positive according to the instructions of the test kit.

### 2.10 Protection against IBDV challenge

Five chickens were randomly selected from each group and weighed at 39 and 42 days of age. Then, they were euthanized and dissected for the bursa. Bursa from chickens was fixed in 4% neutral-buffered formalin for 48 h, embedded in paraffin, and stained with hematoxylin and eosin (H&E). The H&E staining was carried out as previously described (31). Bursa atrophy was evaluated in chickens using the bursa: body weight index (BBIX), and BBIX below 0.70 was considered bursa atrophy (32). Bursa pathology tissue H&E sections were recorded using the histopathological bursa lesion score (HBLS) when HBLS values not greater than 1 (no or minor lesions) were defined as freedom from IBDV attack (33).

### 2.11 Statistical analysis

Statistical analysis was performed using one-way analysis of variance (ANOVA) and Tukey’s multiple comparison test with GraphPad Prism 8.3.0 (GraphPad Software). A *p*-value of <0.05 was considered statistically significant between different groups. All data were presented as the mean ± standard deviation (S.D.).

## 3 Results

### 3.1 Construction of a transgenic *Eimeria acervulina* variant transfected sequentially with two copies of VP2 gene (Ea-2VP2)

To construct a transgenic *E. acervulina* transfected sequentially with two copies of the VP2 gene, we used the Ea-VP2, which carried the VP2 gene and the enhanced yellow fluorescent reporter (EYFP) gene. We constructed a novel single expression-cassette plasmid, pMic-VP2-flag-mCherry-ACT (Figure 1A), in which a VP2 gene and red fluorescent reporter gene (mCherry) were regulated by a microneme 2 promoter of *E. tenella*. After the nucleofection of the Ea-VP2, EYFP and mCherry were detected in a transgenic oocyst *in vitro* by a confocal microscope (Figure 1B). Initially, we obtained a positive population with only 0.11% expressing the mCherry and EYFP genes at the first generation of passage. Then, after four continuous passages in chickens followed by selection with FACS, the population of dual fluorescence reached >95% and remained stable without any additional selection (Table 1).

**Table 1:** Passages of transgenic Ea-2VP2.

Generation	Percentage of fluorescent oocysts (%)	Selection strategy
First^a^	0.11	FACS^b^
Second	43.6	FACS
Third	83.6	FACS
Fourth	95.1	FACS
Fifth–eighth	>95	–^c^

^a^The first generation was obtained by the transfected sporozoites of Ea-VP2 via subwing vein inoculation.

^b^Selection of transgenic Eimeria parasites using fluorescent-activated cell sorting (FACS).

^c^Achieving stability of transgenic Eimeria populations without the need for selection.

To determine whether we established a stable transgenic Ea-2VP2 population, we used PCR with specific primers targeting the VP2 and mCherry genes. This PCR yielded products of the expected size using the genomic DNA of sporulated oocysts (Figure 1C). We used a Western blot assay to detect the expression of the VP2 gene at the sporulated oocyst stage, as shown in Figure 1D, a specific band in the Ea-2VP2 line that is consistent with the sizes of the EaMic2-VP2-flag protein. The results from the indirect immunofluorescence assay (IFA) further evidenced that VP2 protein was expressed and located in the cytoplasm of Ea-2VP2 (Figure 1E). Considering these data together, we successfully obtained an *E. acervulina* population transfected sequentially with two copies of the VP2 gene of IBDV with dual fluorescence reporters, EYFP and mCherry.

### 3.2 Constant expression of VP2 gene through endogenous developmental stage

**Figure 3:**
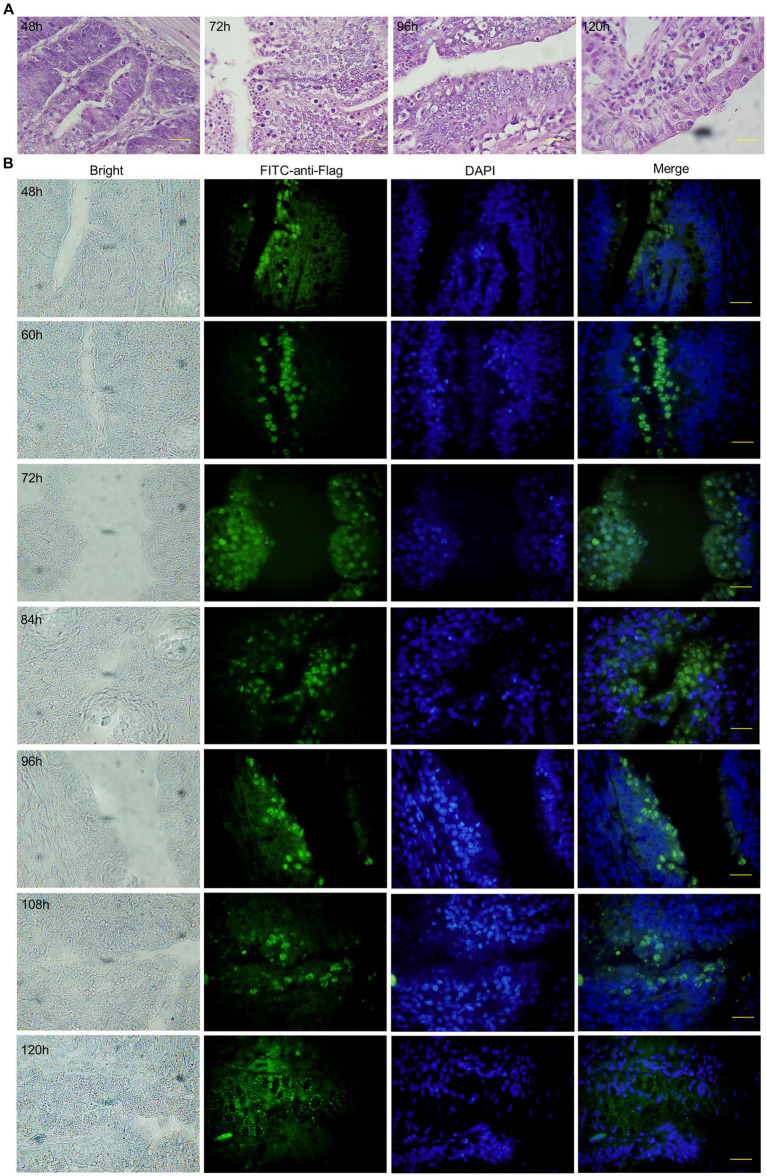
Expression of VP2 at the endogenous stage of Ea-2VP2 in the duodenum. **(A)** H&E staining of duodenum from 48 to 120 h post-inoculation. Bar = 20 μm. **(B)** Immunofluorescence of duodenum from 48 to 120 h post-inoculation. Mouse anti-flag monoclonal (1:200) and FITC-conjugated goat anti-mouse IgG (1:200) were used as the primary and secondary antibodies, respectively. Nuclei were stained with DAPI. Bar = 20 μm.

To detect the expression of the VP2 gene at the endogenous developmental stage of Ea-2VP2 in chickens, we dissected the duodenum of chicks infected with 5 × 10^5^ Ea-2VP2 with an interval of 12 h and observed the H&E tissue sections and tissue immunofluorescence sections of the duodenum. As shown in Figure 3A and Supplementary Figure 1, the developmental stage was detected within a parasitophorous vacuole (PV). The first-generation schizonts were detected in the crypt 48 h post-inoculation (p.i.), and the merozoites and gametocytes were detected in the duodenum villi from 60 h to 120 h p.i., respectively. The expression of EaMic2-VP2-flag was also detected in the merozoites and gametocytes of Ea-2VP2, which was in response to the specific flag tag antibody in the tissue immunofluorescence sections (Figure 3B). These results indicated that the VP2 gene was continually expressed throughout the endogenous developmental stage.

### 3.3 Reproduction of transgenic Ea-2VP2

**Figure 4:**
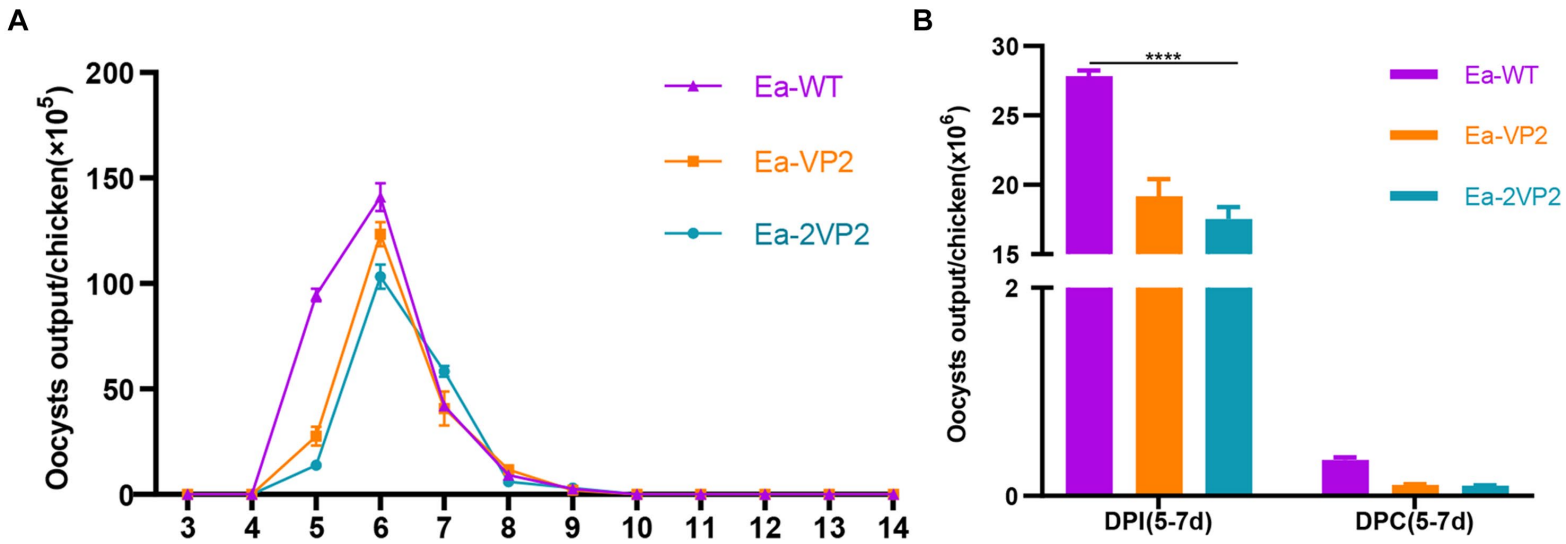
Biological characterization of Ea-2VP2. **(A)** The oocyst output dynamics of Ea-2VP2 were measured every 24 h from days 3 to 14 post-infection (DPI), *n* = 3. **(B)** The total oocyst outputs were measured from days 5–7 days post-infection and days post-challenge (DPC), *n* = 3. (^****^p < 0.0001).

To determine the biological characterization of the oocyst output and immunogenicity, we investigated the oocyst shedding dynamics, the total oocyst output DPI, and DPC. As shown in Figure 4A, the oocyst shedding dynamics of transgenic Ea-2VP2 were similar to those of wild-type *E. acervulina* (Ea-WT), with a peak occurring on day 6 p.i. The total oocyst outputs per chicken inoculated with Ea-2VP2 were significantly lower than those with Ea-WT during 5–7 DPI (*p* < 0.05), which reduced to two-thirds relative to the Ea-WT group (Figure 4B). The total oocyst output DPC of Ea-2VP2 were similar to those of Ea-WT, in which almost no oocysts were discharged during 5–7 DPC (Figure 4B). These results indicated that Ea-2VP2 had less reproductivity than Ea-WT but maintained its high immunogenicity against the wild-type *E. acervulina* challenge.

### 3.4 Ea-2VP2 induced higher primary humoral immune responses than Ea-VP2

**Figure 5:**
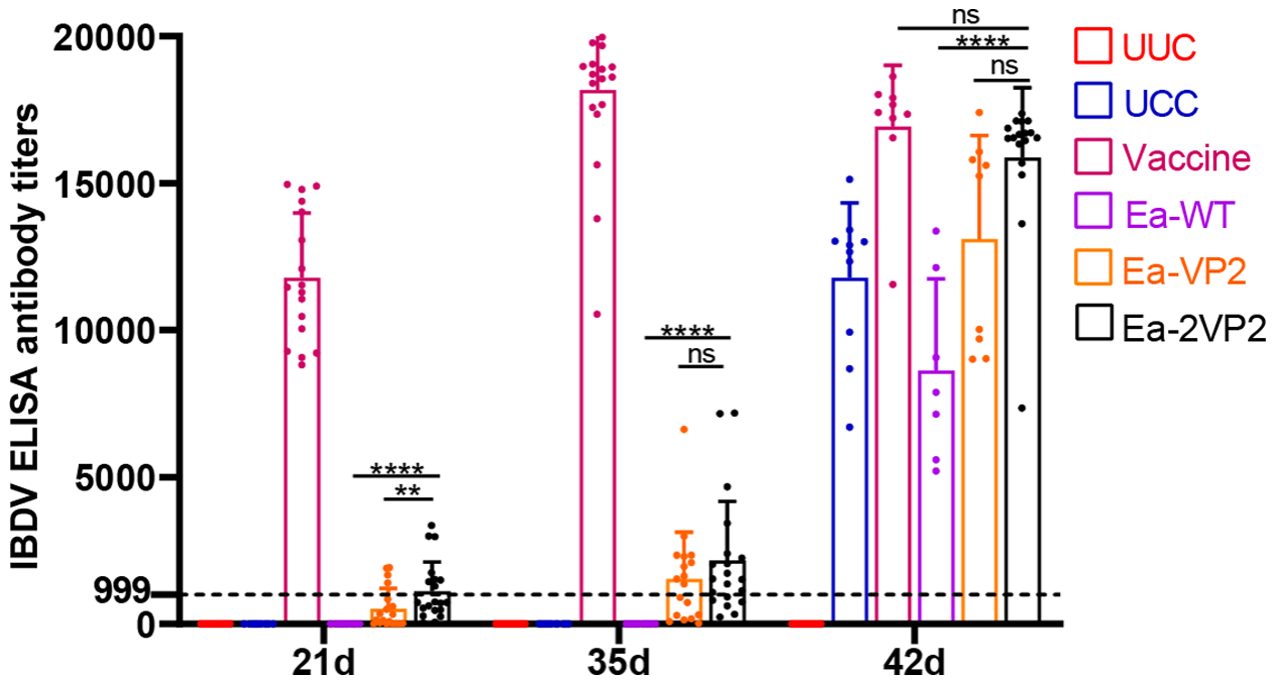
Detection of IBDV antibody titers by ELISA. Serum samples were collected to detect antibody titers using a commercial IBDV antibody test kit (Zoetis, China) at 7, 21, 35, and 42 days of age. Variation in the number of serum samples due to the health status of the chickens, *n* = 7–20 chickens per group per time point (ns: not significant; ^**^p < 0.01; ^****^p < 0.0001).

To detect the levels of humoral immune response after oral immunization, we obtained serum samples to check the specific humoral immune response with blood collected from the subwing vein of chickens following the procedure (Figure 2). The specific antibody titers are shown in Figure 5. The average antibody titers of the Ea-2VP2 group were significantly higher than the Ea-VP2 group on day 14 post-primary immunization, and the mean antibody titers of the groups remained 1,125 and 501, respectively (*p* < 0.05). There was no significant difference between the Ea-2VP2 group and the Ea-VP2 group on day 14 post-secondary immunization and day 7 post-challenge with the 100-fold IBDV B87 strain. The average antibody titers of the Ea-WT group were significantly less than those of the Ea-2VP2 group after two immunizations, which was 0 (*p* < 0.05). Oral immunization of chickens using transgenic parasites was able to induce a specific humoral response in some, but not all, individuals (Figure 5). The above results indicated that increasing the copy number of the VP2 gene expressed could induce a higher primary humoral immune response.

### 3.5 Protective efficacy of Ea-2VP2 to bursal tissue against IBDV

**Figure 6:**
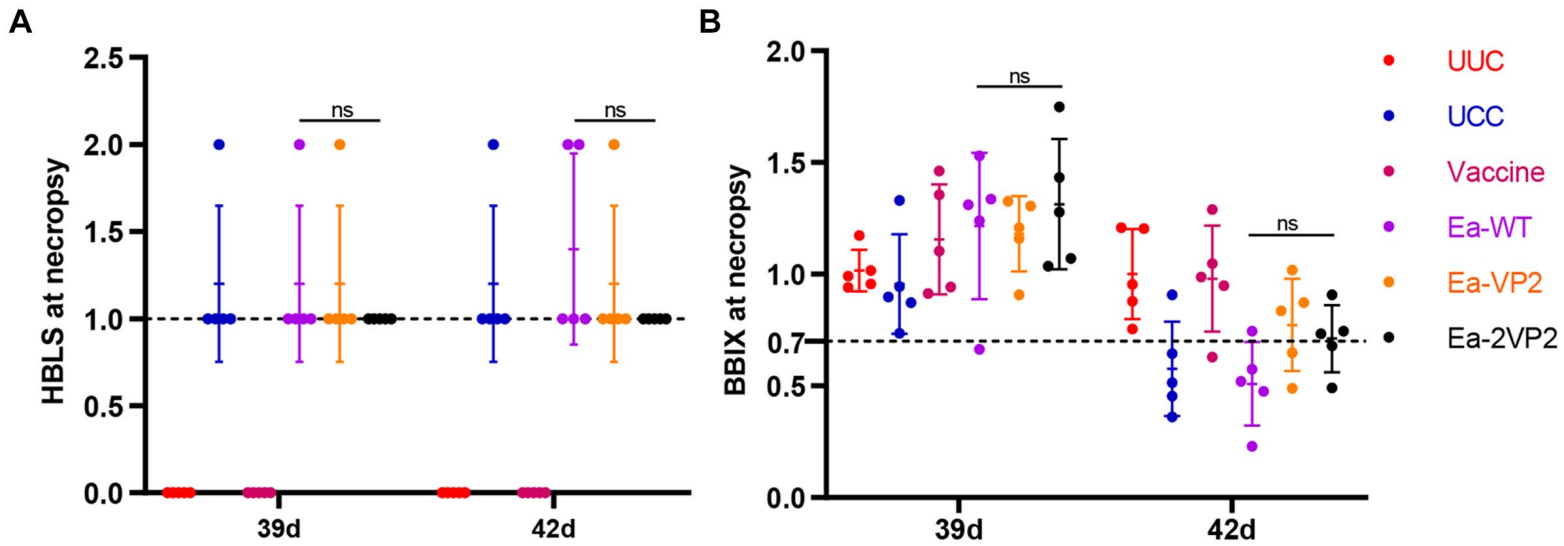
Protective efficacy of transfected Ea-2VP2 against IBDV infection. **(A)** The histopathologic bursal lesion score (HBLS) of chickens at 39 and 42 days of age. 0, no lesions; 1, minor lesions; 2, scattered follicular lesions. When HBLS ≤1, protected; HBLS >1, unprotected. *n* = 5. **(B)** The bursa: body-weight index (BBIX) values of chickens at 39 and 42 days of age, and the BBIX below 0.70 was considered bursa atrophy. *n* = 5. ns, non-significant.

To investigate the protective efficacy of the Ea-2VP2, we randomly selected five vaccinated chickens from each group and weighed them at 39 and 42 days of age after the challenge. Then, chickens were euthanized and dissected for the bursa. The bursa: BBIX and histopathological bursal lesion score (HBLS) were calculated to analyze the extent of bursal atrophy and histopathological lesions. As shown in Figures 6A,B, all the chickens vaccinated with Ea-2VP2 showed an HBLS value of 1. Among the five chickens between the Ea-VP2 and Ea-WT groups, one chicken and two chickens showed mild bursal lesions (HBLS 2), respectively. Among the five chickens in each group, only the Ea-WT group had one chicken with a BBIX value lower than 0.70 on 39 days. The Ea-WT group had four chickens with a BBIX value lower than 0.70, while the Ea-VP2 group and the Ea-2VP2 group had two chickens with a BBIX value lower than 0.70 42 days after the challenge, respectively. The above results indicated that immunizations with Ea-2VP2 appeared to provide a certain degree of protection against bursa damage caused by IBDV infection.

## 4 Discussion

In this research, we successfully established a stable transgenic *E. acervulina* population transfected sequentially with two copies of the VP2 gene of the IBDV known as Ea-2VP2. Oral immunization of chickens using these newly constructed transgenic parasites demonstrated a higher primary humoral immune response against IBDV in certain individuals, although still not higher than the commercial vaccine and not universally. Our investigation revealed that Ea-2VP2 constantly expressed the VP2 gene throughout the endogenous developmental stage in chickens. The Ea-2VP2 produced fewer oocysts than the wild type, and almost no oocysts were discharged after challenge, indicating the maintenance of their immunogenicity against Ea-WT infection. Immunization with Ea-2VP2 showed potential for safeguarding bursal tissue in chickens infected with IBDV. Additionally, the transgenic populations used for the immunizations showed stability in both retention and expression of the exogenous genes.

Notably, oral immunization of transgenic parasites demonstrated the capacity to prime immunity, as evidenced by the fact that the Ea-2VP2 group exhibited more concentrated antibody titers compared to the Ea-VP2 group, both after the primary and secondary immunizations, with a more pronounced effect following the challenge (Figure 5). While increasing the copy number of VP2 genes in transgenic *E. acervulina* seemed like a promising approach to provoking a higher immune response in chickens, the specific antibody levels did not increase double as anticipated. We speculated that this may be related to the proportion of VP2 protein in the transgenic *E. acervulina*, which can be detected by the immune system. The extent to which the VP2 protein content increases proportionally with copy numbers in transgenic *E. acervulina* remains to be tested in further studies.

Previous studies have indicated that the location of VP2 does impact the specific immune response microneme-located VP2 showed stronger immunogenicity than surface-located VP2 (26). Liu et al. constructed transgenic *E. tenella* expressing the monomer M2 protein of the avian influenza virus that could not elicit immune responses (30). Moreover, Zhang et al. constructed a transgenic *E. acervulina* line that expresses 12 copies of M2e, and the immune response was not significantly different from the control group (9). So, the location of immunogen expression may be responsible. The current study has detected antibodies against the VP2 protein using ELISA. However, the immunizations conducted did not align with the prescribed doses for *Eimeria* vaccinations. Various expression systems are available for expressing the VP2 gene. The eukaryotic system, facilitating correct protein folding due to the conformational dependence of antigenic epitopes, renders the expressed VP2 protein more immunogenic compared to the prokaryotic system (34). Co-replication and co-expression of the VP2 gene with viral vectors in the host stimulate an immune response against both viruses. For instance, in a comparative assessment of the herpesvirus of turkey (HVT)-IBD vector with other vaccines against very virulent IBDV (vvIBDV) in broiler chickens, the HVT-IBD vector vaccine demonstrated enhanced safety and provided superior protection against the vvIBDV challenge (35). Insertion of the VP2 gene into the US2 gene locus of Marek’s Disease Virus (MDV) provided complete protection against IBDV and MDV challenge (31). The larger genomes of *Eimeria* accommodate the insertion and expression of multiple foreign antigens in contrast to viral vectors (13). Additionally, transgenic *Eimeria* parasites can be orally administered without incurring additional production costs for delivering their antigens, and they are also deemed safe for meat production as they do not leave behind drug residues derived from anti-coccidial drugs (36). Despite demonstrating the potential of the *Eimeria* expression system in stimulating the humoral immune response, enhancing the immune response to heterologous pathogen antigens remains a challenge.

Over the past two decades, despite the successful development of a transient and stable transfection platform in *Eimeria* parasites, their incapacity to complete the entire life cycle and continuously culture *in vitro* poses obstacles for their genetic manipulation (37). Presently, the selection system used to enhance the fluorescent rate of transgenic parasites primarily involves a combination of FACS and drug selection *in vivo*, owing to the low transfection efficiency (9).

The key challenge in developing *Eimeria* as a vaccine vector lies in improving the immune response to heterologous pathogen antigens. Various strategies have been proposed for this purpose, including but not limited to: (I) increasing the expression of heterologous antigens by augmenting gene copy number or utilizing a more potent promoter in single or multiple expression cassettes (38); (II) enhancing the likelihood of heterologous gene capture by antigen-presenting cells through fusion with cytokines, other molecular adjuvants, or optimizing antigen location (39, 40); (III) modifying the structure of exogenous protein in transgenic *Eimeria* parasites to form virus-like particles (VLPs) (41); and (IV) precisely inserting or integrating exogenous antigens into the immune-dominant antigen of *Eimeria* using the CRISPR/Cas9 technology tool (42, 43).

However, our current detection does not reveal neutralizing antibody levels or cellular immune responses, which are still ongoing. Furthermore, investigating potential synergistic effects between antigens sourced from the virus and *Eimeria* parasites for immune response induction remains necessary. It is of great significance for the development of transgenic *E. acervulina* as a live vector vaccine. Briefly, there remains a long and difficult journey ahead to fully exploit the potential of *Eimeria* parasites for constructing an effective vaccine vector.

## 5 Conclusion

We have achieved a significant step by establishing stable transfection in an *E. acervulina* population transfected sequentially with two copies of the VP2 gene of IBDV with EYFP and mCherry reporter >95%. This successful transfection led to enhanced primary immune responses in chickens. Our findings strongly support the idea that increasing the number of exogenous genes represents a promising strategy for the development of transgenic *Eimeria* parasites serving as a delivery vector for viral antigens.

## Data availability statement

The original contributions presented in the study are included in the article/Supplementary material, further inquiries can be directed to the corresponding authors.

## Ethics statement

The animal study was approved by China Agricultural University Institutional Animal Care and Use Committee. The study was conducted in accordance with the local legislation and institutional requirements.

## Author contributions

QG: Writing – original draft. YY: Data curation, Writing – review & editing. JS: Data curation, Writing – review & editing. XT: Data curation, Project administration, Supervision, Writing – review & editing. SZ: Data curation, Writing – review & editing. CC: Supervision, Writing – review & editing. BB: Supervision, Writing – review & editing. IT: Supervision, Writing – review & editing. XL: Data curation, Project administration, Supervision, Writing – review & editing. GZ: Writing – review & editing, Supervision. XS: Project administration, Writing – review & editing.

## Supplementary material

### SUPPLEMENTARY FIGURE 1

H&E staining of duodenum from 48 to 120 h post-inoculation, bar = 100 μm.

## References

[1] Blake DP Knox J Dehaeck B Huntington B Rathinam T Ravipati V. Re-calculating the cost of coccidiosis in chickens. Vet Res. (2020) 51:115. doi: 10.1186/s13567-020-00837-2, PMID: 32928271 PMC7488756

[2] Gaghan C Adams D Mohammed J Crespo R Livingston K Kulkarni RR. Characterization of vaccine-induced immune responses against coccidiosis in broiler chickens. Vaccine. (2022) 40:3893–902. doi: 10.1016/j.vaccine.2022.05.043, PMID: 35623907

[3] Zaheer T Abbas RZ Imran M Abbas A Butt A Aslam S. Vaccines against chicken coccidiosis with particular reference to previous decade: progress, challenges, and opportunities. Parasitol Res. (2022) 121:2749–63. doi: 10.1007/s00436-022-07612-6, PMID: 35925452 PMC9362588

[4] Attree E Sanchez-Arsuaga G Jones M Xia D Marugan-Hernandez V Blake D. Controlling the causative agents of coccidiosis in domestic chickens; an eye on the past and considerations for the future. CABI Agric Biosci. (2021) 2:37. doi: 10.1186/s43170-021-00056-5, PMID: 34604790 PMC8475900

[5] Blake DP Tomley FM. Securing poultry production from the ever-present *Eimeria* challenge. Trends Parasitol. (2014) 30:12–9. doi: 10.1016/j.pt.2013.10.003, PMID: 24238797

[6] Blake DP Worthing K Jenkins MC. Exploring *Eimeria* genomes to understand population biology: recent progress and future opportunities. Genes. (2020) 11:1103. doi: 10.3390/genes11091103, PMID: 32967167 PMC7564333

[7] Olajide JS Qu Z Yang S Oyelade OJ Cai J. *Eimeria* proteins: order amidst disorder. Parasit Vectors. (2022) 15:38. doi: 10.1186/s13071-022-05159-0, PMID: 35073987 PMC8785600

[8] Jaramillo-Ortiz JM Burrell C Adeyemi O Werling D Blake DP. First detection and characterization of *Eimeria zaria* in European chickens. Vet Parasitol. (2023) 324:110068. doi: 10.1016/j.vetpar.2023.110068, PMID: 37931476

[9] Zhang S Tang X Wang S Shi F Duan C Bi F. Establishment of recombinant *Eimeria acervulina* expressing multi-copies M2e derived from avian influenza virus H9N2. Vaccines. (2021) 9:791. doi: 10.3390/vaccines9070791, PMID: 34358207 PMC8310259

[10] Lee Y Lu M Lillehoj HS. Coccidiosis: recent progress in host immunity and alternatives to antibiotic strategies. Vaccines. (2022) 10:215. doi: 10.3390/vaccines10020215, PMID: 35214673 PMC8879868

[11] Min W Kim WH Lillehoj EP Lillehoj HS. Recent progress in host immunity to avian coccidiosis: IL-17 family cytokines as sentinels of the intestinal mucosa. Dev Comp Immunol. (2013) 41:418–28. doi: 10.1016/j.dci.2013.04.003, PMID: 23583525

[12] Huang J Zhang Z Li M Song X Yan R Xu L. Immune protection of microneme 7 (EmMIC7) against *Eimeria maxima* challenge in chickens. Avian Pathol. (2015) 44:392–400. doi: 10.1080/03079457.2015.1071780, PMID: 26181095

[13] Marugan-Hernandez V Cockle C Macdonald S Pegg E Crouch C Blake DP. Viral proteins expressed in the protozoan parasite *Eimeria tenella* are detected by the chicken immune system. Parasit Vectors. (2016) 9:463. doi: 10.1186/s13071-016-1756-2, PMID: 27553200 PMC4994267

[14] Yan W Liu X Shi T Hao L Tomley FM Suo X. Stable transfection of *Eimeria tenella*: constitutive expression of the YFP-YFP molecule throughout the life cycle. Int J Parasitol. (2009) 39:109–17. doi: 10.1016/j.ijpara.2008.06.013, PMID: 18718473

[15] Clark JD Oakes RD Redhead K Crouch CF Francis MJ Tomley FM. *Eimeria* species parasites as novel vaccine delivery vectors: anti-*Campylobacter jejuni* protective immunity induced by *Eimeria tenella*-delivered CjaA. Vaccine. (2012) 30:2683–8. doi: 10.1016/j.vaccine.2012.02.002, PMID: 22342500

[16] Tang X Liu X Yin G Suo J Tao G Zhang S. A novel vaccine delivery model of the apicomplexan *Eimeria tenella* expressing *Eimeria maxima* antigen protects chickens against infection of the two parasites. Front Immunol. (2018) 8:1982. doi: 10.3389/fimmu.2017.01982, PMID: 29375584 PMC5767589

[17] Pastor-Fernández I Kim S Billington K Bumstead J Marugán-Hernández V Küster T. Development of cross-protective *Eimeria*-vectored vaccines based on apical membrane antigens. Int J Parasitol. (2018) 48:505–18. doi: 10.1016/j.ijpara.2018.01.003 29524526

[18] Trapp J Rautenschlein S. Infectious bursal disease virus’ interferences with host immune cells: what do we know? Avian Pathol. (2022) 51:303–16. doi: 10.1080/03079457.2022.2080641, PMID: 35616498

[19] Yang H Ye C. Reverse genetics approaches for live-attenuated vaccine development of infectious bursal disease virus. Curr Opin Virol. (2020) 44:139–44. doi: 10.1016/j.coviro.2020.08.001, PMID: 32892072

[20] El-Aried TA Mansour SMG ElBakrey RM N Ismail AE Eid AAM. Infectious bursal disease virus: molecular epidemiologic perspectives and impact on vaccine efficacy against avian influenza and Newcastle disease viruses. Avian Dis. (2019) 63:606–18. doi: 10.1637/aviandiseases-D-19-00086, PMID: 31865675

[21] Chen C Qin Y Qian K Shao H Ye J Qin A. HSC70 is required for infectious bursal disease virus (IBDV) infection in DF-1 cells. Virol J. (2020) 17:65. doi: 10.1186/s12985-020-01333-x 32375812 PMC7201719

[22] Ganguly B Rastogi SK. Structural and functional modeling of viral protein 5 of infectious bursal disease virus. Virus Res. (2018) 247:55–60. doi: 10.1016/j.virusres.2018.01.017, PMID: 29427596

[23] Asfor AS Reddy VRAP Nazki S Urbaniec J Brodrick AJ Broadbent AJ. Modeling infectious bursal disease virus (IBDV) antigenic drift in vitro. Viruses. (2022) 15:130. doi: 10.3390/v15010130, PMID: 36680169 PMC9867341

[24] Arnold M Durairaj V Mundt E Schulze K Breunig KD Behrens SE. Protective vaccination against infectious bursal disease virus with whole recombinant Kluyveromyces lactis yeast expressing the viral VP2 subunit. PLoS One. (2012) 7:e42870. doi: 10.1371/journal.pone.0042870, PMID: 23024743 PMC3443089

[25] Maqsood I Shi W Wang L Wang X Han B Zhao H. Immunogenicity and protective efficacy of orally administered recombinant *Lactobacillus plantarum* expressing VP2 protein against IBDV in chicken. J Appl Microbiol. (2018) 125:1670–81. doi: 10.1111/jam.14073, PMID: 30118165 PMC7166448

[26] Yu Y Tang X Duan C Suo J Crouch C Zhang S. Microneme-located VP2 in *Eimeria acervulina* elicits effective protective immunity against infectious bursal disease virus. Infect Immun. (2024) 92:e0045623. doi: 10.1128/iai.00456-23, PMID: 38179959 PMC10863409

[27] Long PL Millard BJ Joyner LP Norton CC. A guide to laboratory techniques used in the study and diagnosis of avian coccidiosis. Folia Vet Lat. (1976) 6:201–17. PMID: 1010500

[28] Tang X Liu X Tao G Qin M Yin G Suo J. “Self-cleaving” 2A peptide from porcine teschovirus-1 mediates cleavage of dual fluorescent proteins in transgenic *Eimeria tenella*. Vet Res. (2016) 47:68. doi: 10.1186/s13567-016-0351-z 27352927 PMC4924277

[29] Liu X Shi T Ren H Su H Yan W Suo X. Restriction enzyme-mediated transfection improved transfection efficiency in vitro in apicomplexan parasite *Eimeria tenella*. Mol Biochem Parasitol. (2008) 161:72–5. doi: 10.1016/j.molbiopara.2008.06.006, PMID: 18606196

[30] Liu X Zou J Yin G Su H Huang X Li J. Development of transgenic lines of *Eimeria tenella* expressing M2e-enhanced yellow fluorescent protein (M2e-EYFP). Vet Parasitol. (2013) 193:1–7. doi: 10.1016/j.vetpar.2012.12.019, PMID: 23298569

[31] Tian Y Xu J Li Y Zhao R Du S Lv C. MicroRNA-31 reduces inflammatory signaling and promotes regeneration in colon epithelium, and delivery of mimics in microspheres reduces colitis in mice. Gastroenterology. (2019) 156:2281–2296.e6. doi: 10.1053/j.gastro.2019.02.023, PMID: 30779922

[32] Li K Liu Y Liu C Gao L Zhang Y Cui H. Recombinant Marek’s disease virus type 1 provides full protection against very virulent Marek’s and infectious bursal disease viruses in chickens. Sci Rep. (2016) 6:39263. doi: 10.1038/srep39263, PMID: 27982090 PMC5159867

[33] Li K Gao L Gao H Qi X Gao Y Qin L. Codon optimization and woodchuck hepatitis virus posttranscriptional regulatory element enhance the immune responses of DNA vaccines against infectious bursal disease virus in chickens. Virus Res. (2013) 175:120–7. doi: 10.1016/j.virusres.2013.04.010, PMID: 23631937

[34] Martinez-Torrecuadrada JL Saubi N Pagès-Manté A Castón JR Espuña E Casal JI. Structure-dependent efficacy of infectious bursal disease virus (IBDV) recombinant vaccines. Vaccine. (2003) 21:3342–50. doi: 10.1016/s0264-410x(02)00804-6, PMID: 12804866

[35] Sedeik ME El-Shall NA Awad AM Abd El-Hack ME Alowaimer AN Swelum AA. Comparative evaluation of HVT-IBD vector, immune complex, and live IBD vaccines against vvIBDV in commercial broiler chickens with high maternally derived antibodies. Animals. (2019) 9:72. doi: 10.3390/ani9030072, PMID: 30813588 PMC6466201

[36] Fatoba AJ Adeleke MA. Transgenic *Eimeria* parasite: a potential control strategy for chicken coccidiosis. Acta Trop. (2020) 205:105417. doi: 10.1016/j.actatropica.2020.105417, PMID: 32105666

[37] Tang X Liu X Suo X. Towards innovative design and application of recombinant *Eimeria* as a vaccine vector. Infect Immun. (2020) 88:e00861-19. doi: 10.1128/IAI.00861-19, PMID: 32094255 PMC7171241

[38] Yin G Liu X Zou J Huang X Suo X. Co-expression of reporter genes in the widespread pathogen *Eimeria tenella* using a double-cassette expression vector strategy. Int J Parasitol. (2011) 41:813–6. doi: 10.1016/j.ijpara.2011.04.001 21550346

[39] Li Z Tang X Suo J Qin M Yin G Liu X. Transgenic *Eimeria mitis* expressing chicken interleukin 2 stimulated higher cellular immune response in chickens compared with the wild-type parasites. Front Microbiol. (2015) 6:533. doi: 10.3389/fmicb.2015.00533, PMID: 26082759 PMC4451583

[40] Tang X Wang C Liang L Hu D Zhang S Duan C. Co-immunization with two recombinant *Eimeria tenella* lines expressing immunoprotective antigens of *E. maxima* elicits enhanced protection against *E. maxima* infection. Parasit Vectors. (2019) 12:347. doi: 10.1186/s13071-019-3605-6, PMID: 31300007 PMC6626336

[41] Mohsen MO Bachmann MF. Virus-like particle vaccinology, from bench to bedside. Cell Mol Immunol. (2022) 19:993–1011. doi: 10.1038/s41423-022-00897-8, PMID: 35962190 PMC9371956

[42] Hu D Tang X Ben Mamoun C Wang C Wang S Gu X. Efficient single-gene and gene family editing in the apicomplexan parasite *Eimeria tenella* using CRISPR-Cas9. Front Bioeng Biotechnol. (2020) 8:128. doi: 10.3389/fbioe.2020.00128, PMID: 32158750 PMC7052334

[43] Tang X Suo J Liang L Duan C Hu D Gu X. Genetic modification of the protozoan *Eimeria tenella* using the CRISPR/Cas9 system. Vet Res. (2020) 51:41. doi: 10.1186/s13567-020-00766-0, PMID: 32160917 PMC7065449

