# Supplementary material for: Oral delivery of Eimeria acervulina transfected sequentially with two copies of the VP2 gene induces immunity against infectious bursal disease virus in chickens
Source: Front Vet Sci. 2024 Apr 10;11:1367912. doi: 10.3389/fvets.2024.1367912 (PMC11041627; doi:10.3389/fvets.2024.1367912)
Supplement: Supplementary file 1 [file Table_1.DOCX]

Table S1. Primers used for the cloning of the different regions of the plasmid.

| Primers | Sequence (5`-3`) |
| --- | --- |
| BB-EtMic2-F | CATGATTACGCCAAGCTGCCCTTAAGCTTCCTAGGATGGAAGATCGC |
| BB-R | AAGGGCAGCTTGGCGTAATCATG |
| VP2-Flag-R | GATCCTTGTAGTCTCCGTCGTGGTCCTTATAGTCCATTCTTCTAATAGCTCTAATAATA |
| P2A-mCh-F1 | TGAAGCAGGCTGGAGACGTGGAGGAGAACCCTGGACCTATGGTGAGCAAGGGCGAGGAG |
| P2A-mCh-F2 | ATGACGATAAGGGAAGCGGAGCTACTAACTTCAGCCTGCTGAAGCAGGCTGGAGACGTG |
| P2A-mCh-F3 | CGACGGAGACTACAAGGATCATGATATTGATTACAAAGACGATGACGATAAGGGAAGCG |
| Ty-R | GTCCAATGGATCCTGATTAGTA |
| Ty-3Actin-F | TACTAATCAGGATCCATTGGACTGAGAATTCGATTGGCCGCGT |
| 3Actin-R | AACCTACAATTACCTGTTGC |
| 3Actin-BB-F | GAAGCAACAGGTAATTGTAGGTTAAGCTTAAGGGCAGCTTCAATTCGCC |
