# Supplementary material for: Oral delivery of Eimeria acervulina transfected sequentially with two copies of the VP2 gene induces immunity against infectious bursal disease virus in chickens
Source: Front Vet Sci. 2024 Apr 10;11:1367912. doi: 10.3389/fvets.2024.1367912 (PMC11041627; doi:10.3389/fvets.2024.1367912)
Supplement: Supplementary file 2 [file Table_2.DOCX]

| Primers | Sequences (5'-3') | |  |
| --- | --- | --- | --- |
| VP2-F | ATGACAAACCTGCAGGATCA |  | |
| VP2-R | TCTTCTAATAGCTCTAATAA |  |  |
| mCherry-F | ATGGTGAGCAAGGGCGAGGA |  | |
| mCherry-R | CTTGTACAGCTCGTCCATGC |  |  |

Table S2 Primers used in identification genomic DNA of Ea-2VP2
