# Supplementary material for: Oral delivery of Eimeria acervulina transfected sequentially with two copies of the VP2 gene induces immunity against infectious bursal disease virus in chickens
Source: Front Vet Sci. 2024 Apr 10;11:1367912. doi: 10.3389/fvets.2024.1367912 (PMC11041627; doi:10.3389/fvets.2024.1367912)
Supplement: Supplementary file 3 [file Table_3.DOCX]

Table S3. Experimental design for animal experiments.

| Groups | Numbers of chickens | Primary immunization per chicken | Second immunization  per chicken | Challenge |
| --- | --- | --- | --- | --- |
| UUC | 20 | / | / | / |
| UCC | 20 | / | / | √ |
| Vaccine | 20 | 300µL | / | √ |
| Ea-WT | 20 | 2×10^4^ | 5×10^5^ | √ |
| Ea-VP2 | 20 | 2×10^4^ | 5×10^5^ | √ |
| Ea-2VP2 | 20 | 2×10^4^ | 5×10^5^ | √ |
