# Supplementary figures and images for: Oral delivery of Eimeria acervulina transfected sequentially with two copies of the VP2 gene induces immunity against infectious bursal disease virus in chickens
Source: Front Vet Sci. 2024 Apr 10;11:1367912. doi: 10.3389/fvets.2024.1367912 (PMC11041627; doi:10.3389/fvets.2024.1367912)

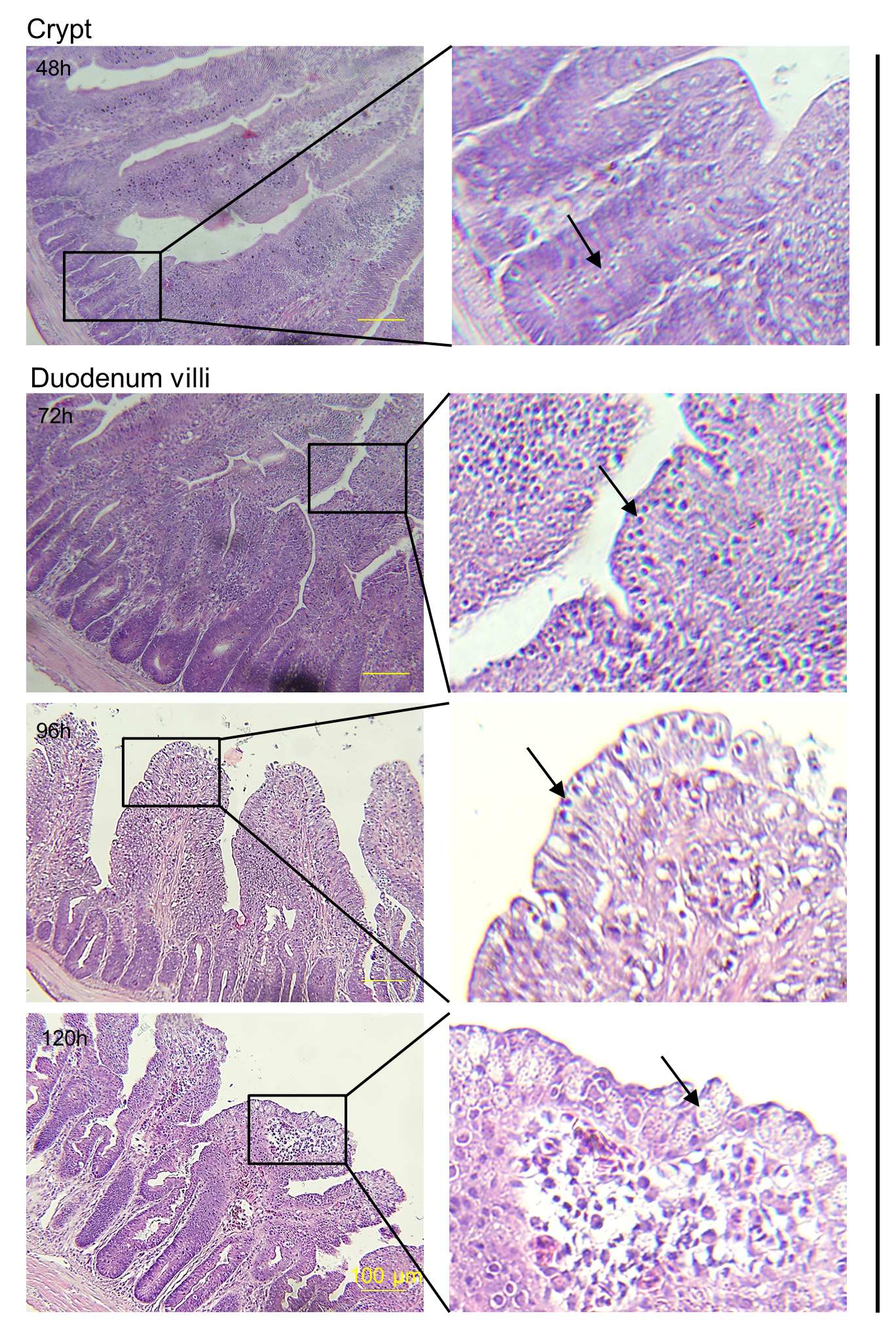

Supplement: Supplementary file 4 [file Image_1.TIF]
